# Ongoing Speciation in the Tibetan Plateau *Gymnocypris* Species Complex

**DOI:** 10.1371/journal.pone.0071331

**Published:** 2013-08-15

**Authors:** Renyi Zhang, Zuogang Peng, Guogang Li, Cunfang Zhang, Yongtao Tang, Xiaoni Gan, Shunping He, Kai Zhao

**Affiliations:** 1 Key Laboratory of Adaptation and Evolution of Plateau Biota, Northwest Institute of Plateau Biology, Chinese Academy of Sciences, Xining, Qinghai, China; 2 University of Chinese Academy of Sciences, Beijing, China; 3 Laboratory of Fish Phylogenetics and Biogeography, Institute of Hydrobiology, Chinese Academy of Sciences, Wuhan, Hubei, China; 4 Key Laboratory of Freshwater Fish Reproduction and Development (Ministry of Education) and Southwest University School of Life Sciences, Beibei, Chongqing, China; Aarhus University, Denmark

## Abstract

Local adaptation towards divergent ecological conditions often results in genetic differentiation and adaptive phenotypic divergence. To illuminate the ecological distinctiveness of the schizothoracine fish, we studied a *Gymnocypris* species complex consisting of three morphs distributed across four bodies of water (the Yellow River, Lake Qinghai, the Ganzi River and Lake Keluke) in the Northeast Tibetan Plateau. We used a combination of mitochondrial (16S rRNA and Cyt *b*) and nuclear (RAG-2) genetic sequences to investigate the phylogeography of these morphs based on a sample of 277 specimens. Analysis of gill rakers allowed for mapping of phenotypic trajectories along the phylogeny. The phylogenetic and morphological analyses showed that the three sparsely rakered morphs were present at two extremes of the phylogenetic tree: the Yellow River morphs were located at the basal phylogenetic split, and the Lake Keluke and Ganzi River morphs at the peak, with the densely rakered Lake Qinghai morphs located between these two extremes. Age estimation further indicated that the sparsely rakered morphs constituted the oldest and youngest lineages, whereas the densely rakered morph was assigned to an intermediate-age lineage. These results are most compatible with the process of evolutionary convergence or reversal. Disruptive natural selection due to divergent habitats and dietary preferences is likely the driving force behind the formation of new morphs, and the similarities between their phenotypes may be attributable to the similarities between their forms of niche tracking associated with food acquisition. This study provides the first genetic evidence for the occurrence of convergence or reversal in the schizothoracine fish of the Tibetan Plateau at small temporal scales.

## Introduction

The discovery of the ecological and evolutionary forces responsible for population divergence and adaptation has long been a major objective of evolutionary biology [1]–[4]. Local adaptation driven by differing ecological conditions often results in the adaptive phenotypic and genetic divergence of geographically isolated populations [1], [5]–[7] and may drive the formation of new taxa [7]–[10]. Such a process requires a source of divergent selection, for which environmental differences and niche adaptation are driving mechanisms [1], [3], [11], [12]. Cases of local adaptation that demonstrate adaptive phenotypic divergence have been well documented in a number of taxa [1], [6], [9], [13]–[15]. However, when two independent evolutionary lineages occupy comparable trophic niches, similar phenotypes can be generated through analogous evolutionary responses [16]–[19]. Evolutionary convergence or reversal in a selected morphological character may then occur [20]–[23]. Schizothoracine fish are specialized for high-elevation rivers and exhibit a number of unique adaptations to the Tibetan Plateau [24], [25]. This large taxon has become an important model for research on speciation in nature and the ecological factors underlying divergence in general [26]–[29]. In this study, we present genetic and morphological evidence of such a genetic divergence in a *Gymnocypris* species complex of schizothoracine fish. And we demonstrate an evidence of ongoing speciation with a tendency to retain the ancestral phenotype on the complex by convergence or reversal.

**Figure 1:**
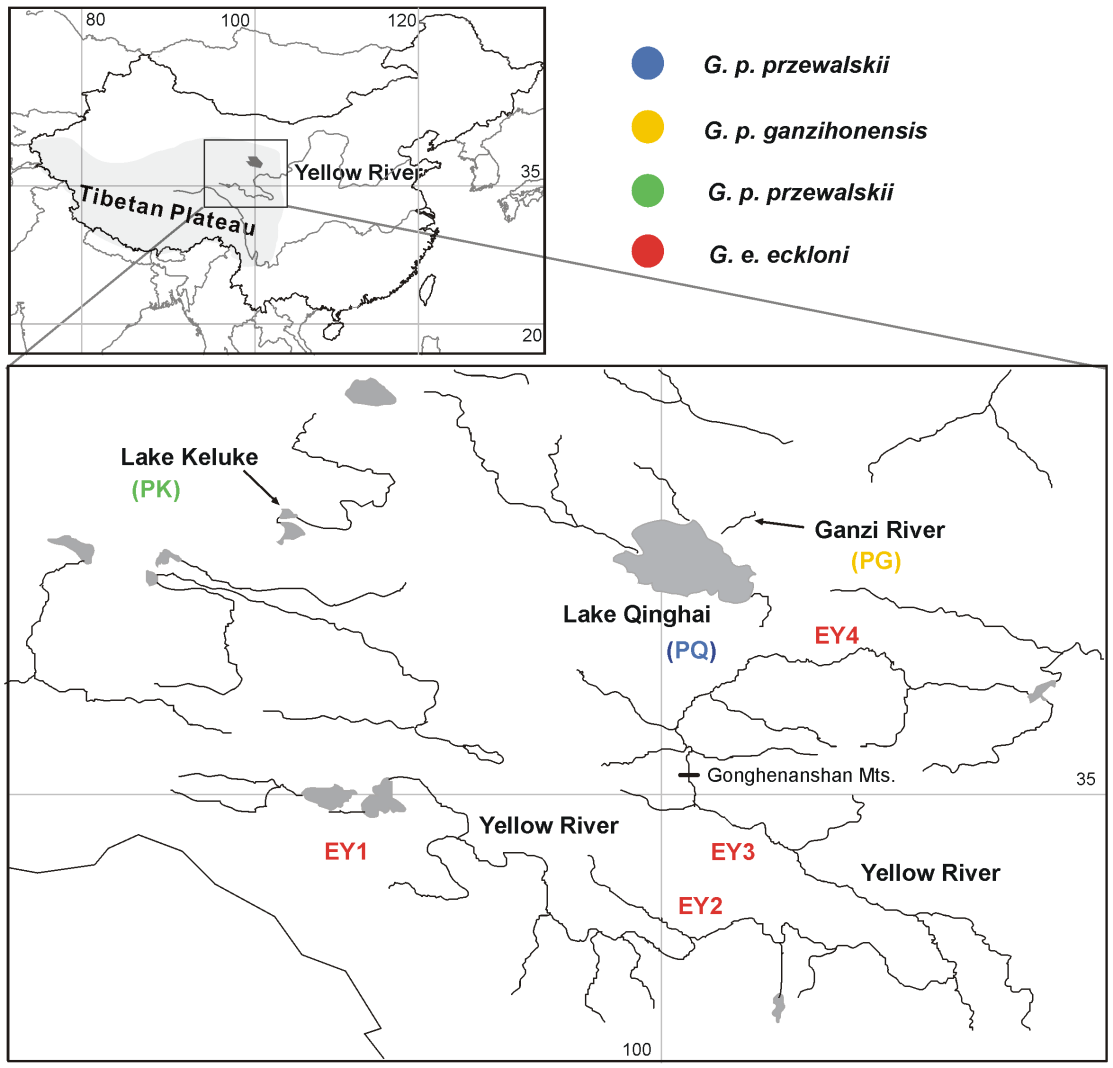
Geographic location of the *Gymnocypris* species complex. This map shows the locations of the samples in the Northeastern Tibetan Plateau and their corresponding codes, as listed in Table 1.

This small species complex is endemic to the Yellow River water system and to the Lake Qinghai water system of the Northeast Tibetan Plateau. The complex includes three members [26], [27], [30], [31]: *Gymnocypris eckloni eckloni*, distributed in the upper reaches of the Yellow River; *Gymnocypris przewalskii przewalskii*, from Lake Qinghai; and *G. p. ganzihonensis*, endemic to the Ganzi River (Fig. 1) [32], [33]. The current speciation hypothesis for these species is that they evolved allopatrically through geographic isolation from an ancestral taxon [32], [34]. This geographical isolation is thought to have occurred at least twice: once through an early series of well-known geological events ca. 15 Ma that caused the separation of Lake Qinghai from the upper reaches of the Yellow River, producing the species *G. przewalskii* (Fig. 1) [34]–[36], and a second, more recent, event caused by the drying of the climate, which separated the Ganzi River from Lake Qinghai and produced the endemic *G. p. ganzihonensis* (Fig. 1) [34].

Local adaptation can result in genetic differentiation and phenotypic divergence, as evidenced by the divergence of gill raker numbers. Gill raker number is associated with food acquisition, and it has been suggested that variations in gill raker number may be at least partially influenced by natural selection [3], [37]–[40]. In the studied *Gymnocypris* species complex, different morphs are often separated by differences in gill raker number, and this trait is assumed to be a reliable marker for assessing the systematic relationships between the species [32], [34]. Interestingly, although *G. e. eckloni* and *G. p. ganzihonensis* evolved in different habitats, they exhibit similar gill raker numbers and morphologies: each bears relatively few, short, sparsely spaced rakers. In contrast, *G. p. przewalskii* is distinctly different, having many long, densely spaced gill rakers [32], [34].

This unique model system has proven to be a good candidate for the study of adaptive convergence or reversal, enabling an evaluation of the relative roles of historical identity, phenotypic distinctness and ecological parameters in the adaptive diversification of the sparsely and densely rakered morphs [3], [38]–[40]. To date, few studies of schizothoracine fish have fully integrated ecological, morphological and life historical information into a genetic framework to address ecological speciation [27], [29]. In this study, we used both mitochondrial and nuclear gene sequences to investigate the evolutionary history of the *Gymnocypris* fish species complex and the possible driving forces underlying its speciation. We examined in detail the phenotypic trajectories of these species along a phylogeny, specifically evaluating whether the speciation of the *Gymnocypris* fish species complex underwent an adaptive convergence or a reversal in gill raker characteristics at small temporal scales.

According to two previous genetic studies of mitochondrial markers, the Yellow River *G. e. eckloni*, Lake Qinghai *G. p. przewalskii* and Ganzi River *G. p. ganzihonensis* form a species complex [26], [27]. The first study (based on Cyt *b*) primarily focused on the phylogeny and population structures of *G. p. przewalskii* and *G. e. eckloni*, only examining a limited number (14) of *G. p. ganzihonensis* individuals. The latter study (based on a control region and Cyt *b*) primarily focused on *G. eckloni* and contained no *G. p. ganzihonensis* individuals. In the present study, we amplified three DNA regions (mitochondrial 16S rRNA, mitochondrial Cyt *b* and nuclear RAG-2) to compare sequence variations among geographic morphs because assessing variations in multiple genes, particularly in both nuclear and mitochondrial genes, generates a more robust inference of genetic structure than assessing a single DNA region [41], [42].

Samples were collected from across the entire range of the complex. In addition, given the high degree of diversification found in this species complex, we considered it likely that collecting populations from a previously unstudied adjacent lake would result in the discovery of new DNA lineages. We therefore sampled individuals from Lake Keluke, which is adjacent to Lake Qinghai. A *Gymnocypris* population has been reported in Lake Keluke [32] and is currently classified as *G. p. przewalskii* [32], although this taxonomic status is not widely accepted. No genetic surveys of this population have been previously conducted due to sampling difficulties; here, we provide the first molecular data for this population.

## Materials and Methods

### Ethics Statement

All necessary permits for collection and experimentation were acquired for the described field study from the Agriculture Department of Qinghai Province, China. All samples of fish used in this study followed the guidelines of the regulations of experiments on animals, and was approved by China Zoological Society. Samples were collected using gill nets or cast nets between January 2011 and December 2012. All the specimens were preserved in 95% ethanol for laboratory analyses.

### Samples and Laboratory Analyses

A total of 277 individuals were obtained from the Yellow River, Lake Qinghai, the Ganzi River and Lake Keluke (Fig. 1; Table 1). *G. e. chilianensis*, *G. waddelli* and *G. potanini*, three species closely related to *G. e. eckloni*, were chosen as outgroups. Voucher specimens were deposited at the Northwest Plateau Institute of Biology, in the Chinese Academy of Science in Xining.

**Table 1:** Sampling locations, sample sizes (*N*) and water systems for each taxon in this study.

Species	*N*	Code	Sampling location	Water system
*G. p. przewalskii*	86	PQ	Qinghai Lake, Qinghai	Lake Qinghai
*G. p. ganzihonensis*	63	PG	Ganzi River, Qinghai	Lake Qinghai
*G. p. przewalskii*	51	PK	Keluke Lake, Qinghai	Qaidam River
*G. e. eckloni*	22	EY1	Maduo, Qinghai	Yellow River
*G. e. eckloni*	17	EY2	Guoluo, Qinghai	Yellow River
*G. e. eckloni*	17	EY3	Hainan, Qinghai	Yellow River
*G. e. eckloni*	21	EY4	Guide, Qinghai	Yellow River

The location codes correspond to those in Fig. 1.

Total genomic DNA was extracted from muscle tissue using a phenol/chloroform extraction procedure [43]. A complete sequence of the Cyt *b* gene and partial sequences of the 16S rRNA and RAG-2 genes were obtained for all the sampled individuals. The primer information is listed in Table 2. The polymerase chain reaction (PCR) mixture contained approximately 100 ng of template DNA, 1 µL of each primer, 5 µL of 10× reaction buffer, 2 µL of dNTPs (each 2.5 mM) and 2.0 U of Taq DNA polymerase, in a total volume of 50 µL. The PCR conditions included an initial denaturation at 94°C for 3 min; 30 cycles of denaturation at 94°C for 1 min, annealing at 58–64°C for 1 min and extension at 72°C for 1 min; and a final extension at 72°C for 5 min. The amplified DNA was fractionated by electrophoresis through 0.8% low-melting agarose gels, recovered from the gels and purified with Gel Extraction Mini kit (Watson Biotechnologies, Shanghai, China). The purified DNA was sequenced on an ABI 3730 capillary sequencer with the Perkin-Elmer BigDye DNA Sequencing Kit according to the manufacturer’s protocol, using the primers previously employed in the PCR (Beijing Tianyi Huiyuan Bioscience and Technology Incorporation, Beijing, China). Newly reported sequences in this paper have been deposited in GenBank under accession numbers KC733894-KC734002, KC76749-KC767659 and KC757127 (see Appendix S1).

**Table 2:** Primers used for PCR and sequencing.

Locus	Primer name	Primer sequence	Primer reference
Cyt *b*	L14724	5′-GAC TTG AAA AAC CAC CGT TG-3′	[69]
	H15915	5′-CTC CGA TCT CCG GAT TAC AAG AC-3′	[69]
16S rRNA	16Sp1F	5′-CTT ACA CCG AGA ARA CAT C-3′	[70]
	16Sp1R	5′-CTT AAG CTC CAA AGG GTC-3′	[70]
RAG-2	RAG2-f2	5′-ARA CGC TCM TGT CCM ACT GG-3′	[71]
	RAG2-R6	5′-TGR TCC ARG CAG AAG TAC TTG-3′	[71]

### Phylogenetic Analyses

The sequences were initially aligned using the program CLUSTALX 1.8 [44], with further corrections conducted by visual analysis. Concordance of the three genes used to construct the data sets was evaluated with the partition homogeneity test implemented in PAUP 4.0b10a [45]. The phylogenetic analyses of the three-region concatenation were reconstructed using the neighbor-joining (NJ) and maximum parsimony (MP) approaches in PAUP 4.0b10a [45], and the Bayesian inference (BI) of likelihood was implemented using MrBayes version 3.1 [46], with different parameter estimations for each of the three DNA regionsjavascript:void(0). The most appropriate model of DNA substitution (GTR+I+G) identified by ModelTest 3.7 [47] was implemented in the NJ and BI analyses. To assess the statistical significance of the hypothesized lineages, a bootstrap analysis with 1,000 replicates was performed for both the NJ and MP analyses. For the BI approach, the best-fitting nucleotide substitution models (GTR+I+G) for the three DNA regions were selected using the Akaike Information Criterion (AIC) as implemented in ModelTest 3.7 [47]. The posterior distributions were obtained by a Markov Chain Monte Carlo (MCMC) analysis with one cold chain and three heated chains. The maximum likelihood model employed six substitution types (*N*_ST_ = 6), with the proportion of sites being invariant (rates = propinv). Samples of the trees and parameters were drawn every 100 steps from a total of one million MCMC generations. We performed three additional analyses beginning with random trees. The consensus of the post-burn for all the generations was computed from all four runs. Furthermore, we constructed median-joining networks using the program Network version 4.5 [48] to visualize the relationships among the haplotypes within these lineages.

### Demographic History

Mismatch distributions and Fu’s *F*s test [49] were conducted using Arlequin version 3.5 [50]. The moment estimator of the time to expansion (τ) was computed, and the time of the main expansion in generations (*t*) was estimated by the equation τ = 2*ut* [51]–[54], with *u* representing the mutation rate per sequence and per generation. The value of *u* was calculated using *u* = 2 µ*k*, with µ representing the mutation rate per nucleotide and *k* representing the number of nucleotides in the analyzed fragment. Finally, the approximate time of expansion in years was calculated by multiplying *t* by the generation time (4 years; [55]) of the schizothoracine fish. We used the previously established average substitution rate of 1.69% per million years [27], which was calibrated for the mitochondrial DNA of the schizothoracine fish.

### Analyses of Genetic Structure and Gill Raker Numbers

The nucleotide diversity (*π*) indices [56] and pairwise genetic differentiation (*F*_ST_) values [57], [58] were calculated using Arlequin version 3.5 [50]. The overall mean divergence among haplotypes and the net divergence between lineages were calculated using MEGA3 software [59], and standard errors were estimated by bootstrapping with 10,000 replicates. To determine the associations between gill raker number and genetic structure, we counted the outer rakers and inner rakers of the first gill arch for all 277 sampled individuals, a decisions based on the morphological characteristics of the different morphs [32], [34].

## Results

### Sequence Information

We sequenced the Cyt *b*, 16S rRNA and RAG-2 genes of 280 individuals, including the three outgroup specimens. The alignment performed for the ingroup individuals revealed 43 variable sites in the Cyt *b* sequences (1140 base pairs [bp]), 25 of which were parsimony informative. The 16S rRNA gene was 1,118 bp in length and included 22 variable sites, 14 of which were parsimony informative. The RAG-2 sequence was 894 bp in length, with 28 variable sites, 14 of which were parsimony informative. No stop codons, insertions or deletions were found in any of the sequences.

Only 43, 22 and 34 unique haplotypes were defined for the Cyt *b*, 16S rRNA and RAG-2 genes, respectively; therefore, we combined the sequences from the three DNA regions to assess variation. Combined mitochondrial DNA regions have proven useful for elucidating both inter- and intraspecific relationships among the schizothoracine fish [27], [28], [31]. A partition homogeneity test indicated the absence of significant incongruence among the Cyt *b*, 16S rRNA and RAG-2 genes (*P*>0.05). This apparent phylogenetic congruence justified the combination of the three partial sequences into a single, 3152-bp fragment for the phylogenetic analysis. A total of 113 haplotypes were defined, including three from the outgroup individuals (see Appendix S1).

### Haplotype Distribution and Genetic Diversity

**Figure 2:**
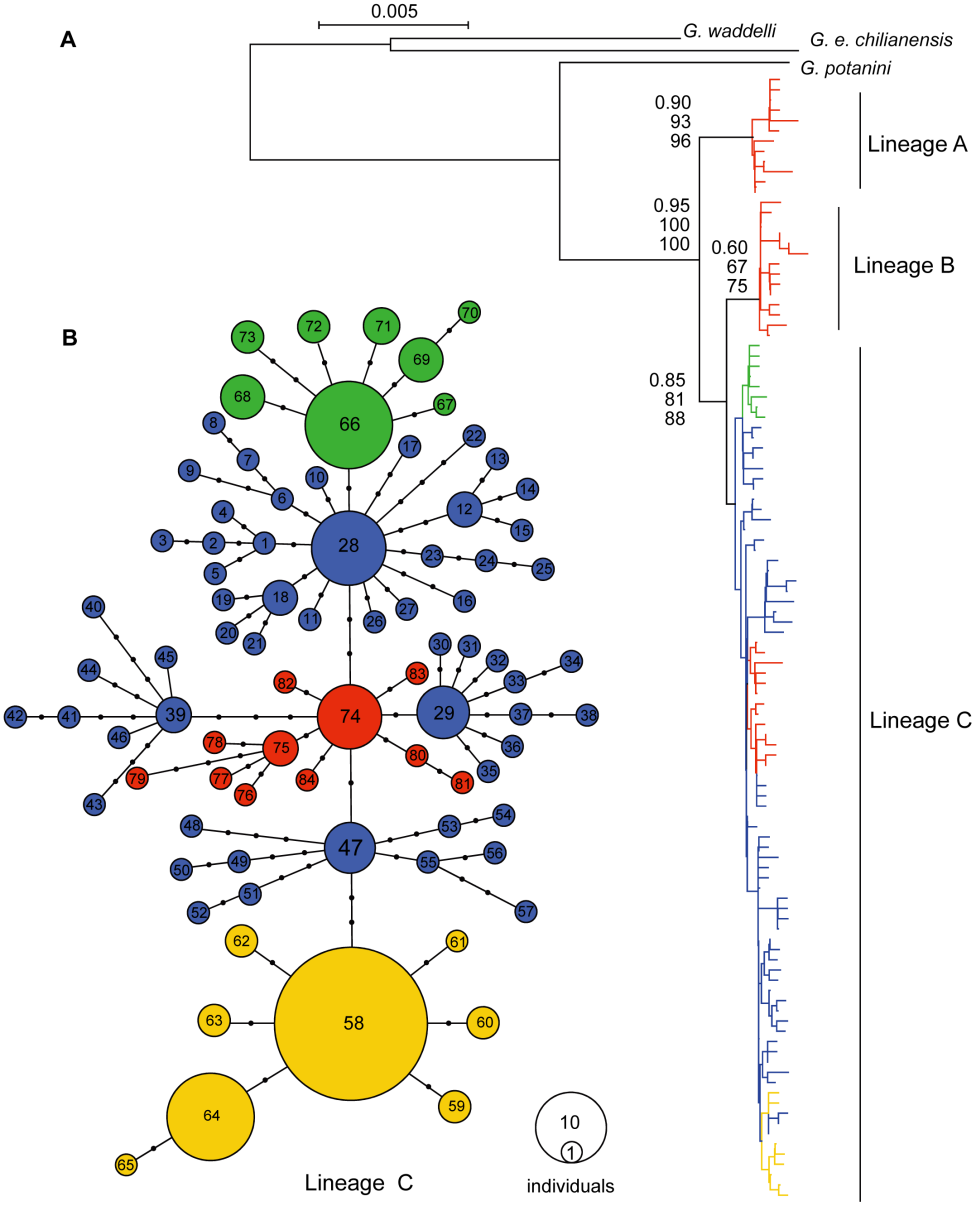
Phylogenetic analyses of the *Gymnocypris* species complex. **A)** The neighbor-joining tree (GTR+I+G model) obtained by combining the Cyt *b*, 16S rRNA and RAG-2 genes (3,152 bp) of all haplotypes defined in the present study. The geographic origins of the haplotypes are illustrated by the color codes used in Fig. 1. The numbers on the branches correspond to a bootstrap support of ≥60% obtained in the BI, NJ and MP analyses. **B)** The median-joining network based on the combined sequence data for all the haplotypes assessed in the present study. The haplotype numbers correspond to those in the Appendix S1. The circle sizes represent the approximate numbers of individuals, and the scale is provided in the lower right corner. The black dots indicate the nucleotide substitutions inferred for that branch. The numbers of mutational steps joining the clades are indicated along the connecting branches. Lineage B was linked to lineage C via haplotype 74 by the smallest number of mutations (five mutations; data not shown). The geographical origins of the haplotypes are illustrated by the same colors used in Fig. 1.

No haplotypes were shared between the three morphs (*G. p. przewalskii*, *G. p. ganzihonensis* and the Yellow River *G. e. eckloni*). Each haplotype was present in only one of the river systems (Fig. 2; Appendix S1).

The nucleotide diversity was highest in the Yellow River *G. e. eckloni* (π = 0.0022±0.0011), lowest in the Ganzi River *G. p. ganzihonensis* and the Lake Keluke *G. p. przewalskii* (π = 0.0003±0.0001 and 0.0002±0.0001, respectively) and intermediate in the Lake Qinghai *G. p. przewalskii* (π = 0.0012±0.0007). The estimated maximum divergences between the haplotypes were 0.26% for the Yellow River *G. e. eckloni*, 0.19% for the Lake Qinghai *G. p. przewalskii*, 0.07% for the Lake Keluke lineage and 0.06% for the Ganzi River lineage.

### Phylogenetic Analyses

All three phylogenetic methods (NJ, MP and BI) used for the 113 haplotypes obtained from the combined data resulted in trees with similar topologies. We found evidence of three distinct lineages (lineages A, B and C; Fig. 2A) rather than two taxonomic species (*G. przewalskii* and *G. eckloni*) or three subspecies (*G. p. przewalskii*, *G. p. ganzihonensis* and *G. e. eckloni*). A clear branching order for the Yellow River *G. e. eckloni* individuals was found in each of the three lineages, whereas the branching of *G. p. przewalskii* and *G. p. ganzihonensis* occurred only in lineage C (Figs. 2A, 2B). The divisions of these lineages were well supported (81–100%) and showed strong geographical associations. Lineages A and B both corresponded to the Yellow River *G. e. eckloni*, whereas the haplotypes of lineage C were widely distributed in both *G. p. przewalskii* from Lake Qinghai and Lake Keluke and *G. p. ganzihonensis* from the Ganzi River, as well as in *G. e. eckloni* from the Yellow River. The mean sequence divergence between lineage A and the other lineages was 0.66% ±0.09, and the net divergence was 0.37% ±0.08; the mean sequence divergence between lineages B and C was 0.38% ±0.07, with a net divergence of 0.19% ±0.07.

The haplotype network for lineage C exhibited seven clusters of haplogroups (haplogroups 28, 29, 39, 47, 58, 66 and 74; Fig. 2B). Although each pair of haplogroups was separated by no more than three mutations, each haplogroup was clearly arrayed in a star-like structure around a central common haplotype (Fig. 2B). These seven haplogroups corresponded to four geographically defined bodies of water. Haplogroups 28, 29, 39 and 47 contained only samples from the Lake Qinghai *G. p. przewalskii*. Haplogroup 66 was composed of only the Lake Keluke *G. p. przewalskii*, whereas haplogroup 58 was restricted to the *G. p. ganzihonensis* endemic to the Ganzi River. The remaining haplogroup, 74, comprised only samples of *G. e. eckloni* from the Yellow River. Lineage B was linked to lineage C via haplotype 74 by the smallest number of mutations (five mutations; data not shown).

### Demographic History

**Figure 3:**
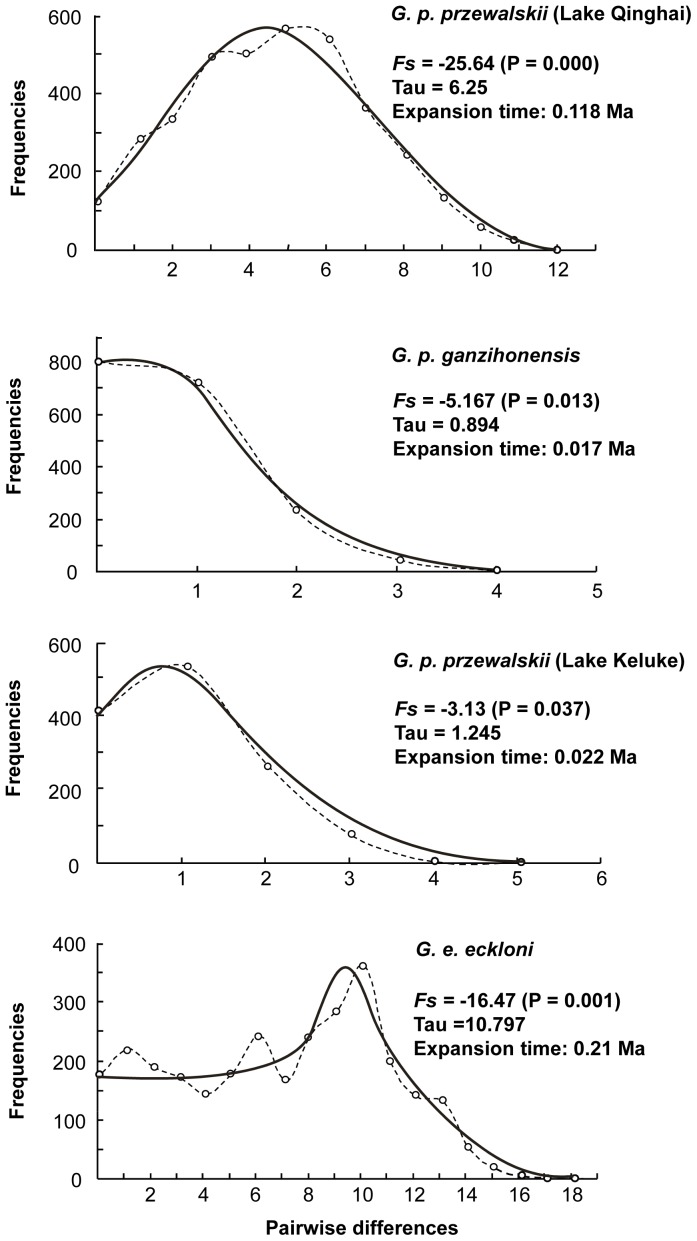
Mismatch distributions of *Gymnocypris* individuals based on the combined sequence data. The observed frequency is indicated by a dotted line. The expected distribution is represented by a continuous line. The values of Fu’s *F*s with *P*-values, τ and the expansion time are shown.

Unimodal mismatch distributions and significant negative values for Fu’s *F*s were observed for *Gymnocypris* individuals from the four bodies of water (Fig. 3). These results suggest the occurrence of expansion events in the demographic histories of these populations. The earliest recent expansions were estimated to have occurred approximately ca. 0.017–0.21 Ma, as shown in Fig. 3.

### Associations between Gill Raker Numbers and Genetic Structure

**Figure 4:**
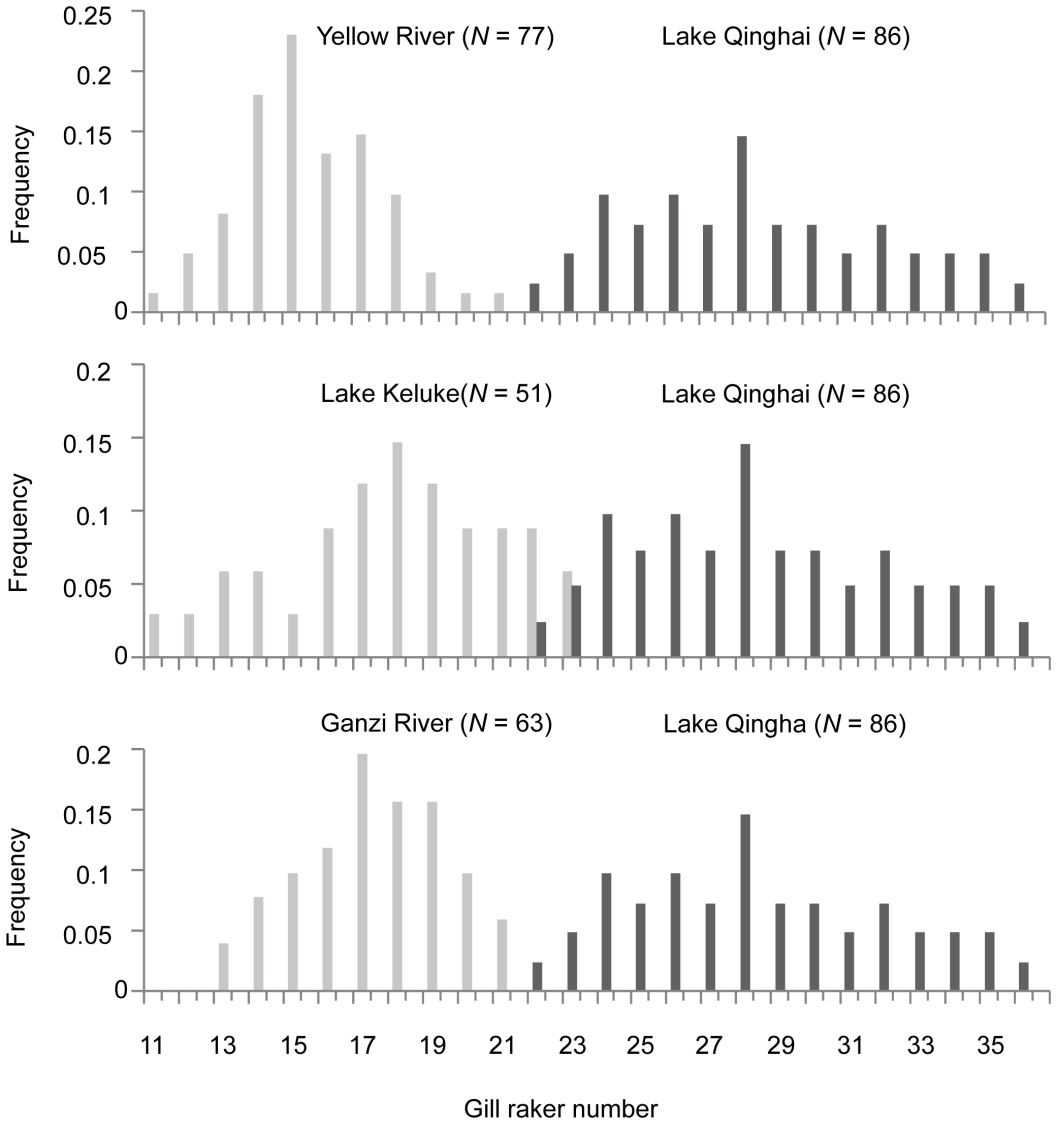
A comparison of the gill raker number distribution in *Gymnocypris* individuals from different bodies of water. The densely rakered morph in Lake Qinghai were compared to the sparsely rakered Yellow River *G. e. eckloni*, Ganzi River *G. p. ganzihonensis* and Lake Keluke *G. p. przewalskii*, respectively. Sample size (*N*) is given.

The *Gymnocypris* fish of all four populations were characterized by a bimodal frequency distribution of gill raker number (Fig. 4), which was composed of three sparsely rakered morphs and one densely rakered morph (Fig. 4). The gill raker numbers were similar among the sparsely rakered *Gymnocypris* morphs from the Yellow River, Lake Keluke and the Ganzi River (with average outer/inner raker numbers of 15.31/21.47, 17.91/30.71 and 17.59/26.3, respectively) and among the densely rakered morphs from Lake Qinghai (with average outer/inner raker numbers of 28.09/44.57) (Table 3). The sparsely and densely rakered morphs showed highly significant differences in these measures (t-test: *P*<0.01).

**Table 3:** Morphological and ecological characteristics of the *Gymnocypris* morphs in this study.

Taxon	Average rakers on the first gill		*N*	Habitats^a^	Main food types^a^
	Outer rakers	Inner rakers			
*G*. *e*. *eckloni*(Yellow River)	15.31±1.850	21.47±3.027	77^b^	Lake or river, deep waters; freshwater	Zoobenthos, plankton, algae, hydrophytes, small fish
	16.88±3.036^a^	23.96±4.280^a^	90		
*G*. *p. przewalskii*(Lake Qinghai)	28.09±4.017	44.57±4.599	86	Brackish-water lake	Plankton, algae
	29.50±5.043^a^	45.50±6.827^a^	701		
*G*. *p. przewalskii*(Lake Keluke)	17.91±3.175	30.71±6.798	51	Brackish-water lake; rare	Benthic organisms, gammarids, chironomid larvae
*G*. *p. ganzihonensis*(Ganzi River)	17.59±2.407	26.30±2.686	63	River; freshwater; rare	Benthic organisms, algae
	17.90±2.390^a^	28.20±3.500^a^	225		

a Data from Tsao & Wu [55], Zhu & Wu [34], Wang [67], Zhao *et al*. [68], Wu & Wu [32] and Qin *et al*. [72].

b The sample size consist of four sampling locations which were given in Table 1.

Pairwise comparisons of genetic differentiation (*F*_ST_) based on the combined data revealed strong differences among the *Gymnocypris* fish from each of the four environments (Table 4). A high level of differentiation was detected between the three sparsely rakered morphs (the Yellow River, Lake Keluke and Ganzi River morphs), whereas the densely rakered Lake Qinghai morph exhibited only an intermediate level of differentiation from any of the other morphs (Table 4). The *F*_ST_ test results were in agreement with the results of the phylogenetic analyses.

**Table 4:** *F*st values among species or populations based on the combined sequence data.

Species	Code	PQ	PG	PK
*G. p. przewalskii*	PQ			
*G. p. ganzihonensis*	PG	0.4654**		
*G. p. przewalskii*	PK	0.3658**	0.6754**	
*G. e. eckloni*	EY	0.3083**	0.5491**	0.5281**

Significant pairwise differences:

** *P*<0.01. The location codes correspond to those used in Fig. 1 and Table 1.

## Discussion

### Phylogeographic Processes and Age Estimation

Lake Qinghai and the Yellow River water system together represent the clearest geologically based barrier to fish movement, and the key aspects of this area’s geological history are well documented. Lake Qinghai is the largest brackish water lake in the Tibetan Plateau. This lake originated due to tectonic activity, and its complete closure has been dated to approximately 0.15 Ma [36], [60], [61]. Originally, the lake was connected to the ancient Yellow River [35], [36], [60], [61]. The “Gonghe Movement” event of the Tibetan Plateau in the Late Pleistocene led to the uplift of the present barrier, and the single body of water was separated into its current configuration of two large water systems (Fig. 1) [36], [60]–[62]. Lake Qinghai was completely closed, and the Yellow River began to cut through the Gonghenanshan Mountains and captured further ancient limnetic basins, reaching upward to its present headwaters. This event offers sufficient support for a Late Pleistocene origin of *G*. *p. przewalskii* from the Yellow River *G. eckloni* [34]. More recently, the Ganzi River was separated from Lake Qinghai by a water level drop, which led to the transformation of the lower reaches of the Ganzi River into a subterranean river that now flows into Lake Qinghai (Fig. 1). As a result, *G. p. przewalskii* evolved into the endemic *G. p. ganzihonensis* within an isolated environment [34]. There are no detailed data on the origin of the fish in Lake Keluke, but the stratigraphic evidence indicates that the lake's two largest tributaries and those of Lake Qinghai were adjacent and that both derived from the northern Qilian Mountains, which exhibit a recent stratigraphic uplift that may have promoted the isolation of the two lakes [63].

We discovered two lineages (A and B) of the Yellow River *G. e. eckloni* at the basal phylogenetic split of this species complex and one lineage (C) that was shared among all of the *Gymnocypris* individuals from Lake Qinghai, Lake Keluke and the Ganzi River (Fig. 2a, b). This branching order implies that the genetic differentiation of the Yellow River *G. e. eckloni* may have existed long before Lake Qinghai separated from the Yellow River. These observations suggest that three lineages of the Yellow River *G. e. eckloni* may have originated from an ancestral population that fragmented into isolated populations and that the ancient lake basins in the limits of the upper Yellow River might have acted as evolutionary reservoirs for the fish.

Our haplotype network approach enabled a fine-grained reconstruction of the evolutionary history of the young lineage C, showing through a network tree approach that the Yellow River *G. e. eckloni* should be considered ancestral to lineage C. First, the central predominant haplotype (haplotype 74; Fig. 2B) was composed entirely of Yellow River *G. e. eckloni*. Second, haplotype 74 was linked to the older lineage B through the smallest number of mutations (five mutations, data not shown). Third, at least four lineages derived from haplotype 74 have seeded Lake Qinghai to form the *G*. *p*. *przewalskii* population. It is apparent that the Yellow River *G. e. eckloni* was crucial to the evolution of the *Gymnocypris* fish in this complex.

The discovery of two well-defined groups endemic to the Ganzi River and Lake Keluke, respectively, was surprising. This result demonstrates the usefulness of sampling a crucial population from Lake Keluke and of utilizing combined sequence data, particularly those from both nuclear and mitochondrial DNA. The *Gymnocypris* fish from the Ganzi River and Lake Keluke formed two monophyletic groups (haplogroups 58 and 66, respectively) that were sisters to two *G. p. przewalskii* lineages (haplogroups 47 and 28, respectively). It is also apparent that the Lake Qinghai *G. p. przewalskii* played a critical role in the evolution of the *Gymnocypris* fish of the Ganzi River and Lake Keluke. A clear pattern of decreasing genetic diversity was detected among the four bodies of water, and the age estimation suggested that the most recent demographic expansion occurred in the two newer morphs (Fig. 3). Together with the geological evidence, these data indicate that a sequential process of adaptive divergence occurred in this *Gymnocypris* species complex.

### Ecological Speciation with a Tendency toward Convergence or Reversal in Gill Raker Traits

The three phylogenetic lineages (A, B and C) were not associated with operational taxa. This lack of congruence between the genetic and phenotypic patterns calls into question the validity of gill raker number as a marker of taxonomic separation. However, gill raker number may still be valuable for identifying ecological distinctiveness and evolutionary forces [40], [64].

*Gymnocypris* fish (three sparsely rakered morphs and one densely rakered morph) in the four environments were characterized by a bimodal frequency distribution of gill raker number with very few overlapping individuals. The densely rakered Lake Qinghai *G. p. przewalskii* fish were characterized mainly by their many long gill rakers, in contrast to the sparsely rakered Yellow River *G. e. eckloni*, Ganzi River *G. p. ganzihonensis* and Lake Keluke *G. p. przewalskii*, which had fewer, shorter rakers (Table 3). However, this study demonstrated that the three sparsely rakered lineages were located at two extremes of the phylogenetic tree: the Yellow River *G*. *e*. *eckloni* was located at the basal phylogenetic split, and the Lake Keluke *G*. *p*. *przewalskii* and Ganzi River *G*. *p*. *ganzihonensis* were at the peak, with the densely rakered Lake Qinghai *G*. *p*. *przewalskii* clearly located between the two extremes (Fig. 2AB). These results suggest that the similar morphologies of the three sparsely rakered morphs may have originated through a complex mechanism, such as introgressive hybridization, retention of ancestral polymorphism or parallel or convergent evolution triggered by directional selection toward similar morphology [65]. The recently derived morphs with sparse rakers formed two monophyletic groups (haplogroups 58 and 66), and each was fully geographically separated from the other, making an introgressive hybridization scenario unlikely. The hypothesis of ancestral polymorphism is more difficult to exclude, but morphological data have demonstrated that *G. p. ganzihonensis* has a distinct lower jaw horn, which is absent in *G. e. eckloni* [32], [34]. This observation may indicate that the sparsely rakered trait in these two species originated through independent evolution along different trajectories. This leaves convergence or reversal in the gill raker number trait as the most likely mechanism. Support for this hypothesis includes the detection of a high level of differentiation (*P*<0.01) among the three sparsely rakered morphs (Table 4). The phylogeographic analyses and age estimation further indicated that the sparsely rakered morphs constituted the oldest and youngest lineages, with the densely rakered morph assigned to an intermediate type.

The evolutionary trends in gill raker number have also been associated with potentially causal ecological factors [3]. Typically, fish with densely rakers prefer a plankton diet, whereas sparse rakered morphs primarily consume zoobenthos [3], [37]–[40], [66]. The character of trophically relevant structures is tightly linked to the ability of schizothoracine fish to process alternative food types [55]. In this *Gymnocypris* species complex, gill raker number is also associated with food acquisition, and the observed feeding preferences are consistent with ecological specialization (Table 3) [34], [67]. The Lake Keluke *G. p. przewalskii* and the Ganzi River *G. p. ganzihonensis* have been found to feed primarily on benthic organisms, such as mud-dwelling gammarid and chironomid larvae, and occasionally on algae attached to stones [68]. The Lake Qinghai *G. p. przewalskii* have been reported to prefer the consumption of plankton, such as copepoda and bacillariophyta [34], [67]. In contrast, the Yellow River *G. e. eckloni* have a wider niche corresponding to a variety of food items, with a preference for zoobenthos and a tolerance for plankton and algae as alternative food sources (Table 3) [32], [55], which is consistent with the usual location of the Yellow River *G. e. eckloni* in deeper waters. Geographically localized natural selection can drive local adaptation and often results in the adaptive phenotypic and genetic divergence of populations [1], [6]–[10]. Therefore, for the *Gymnocypris* fish in this study, disruptive natural selection due to divergent habitats and dietary preferences is the most likely driving force underlying the observed divergence.

Although these variable environments may have promoted rapid divergence, changes in these environments may lead to rapid convergence or the reversal of divergence [22]. Our data suggest that evolution has sometimes favored the same trait combinations and food type changes may have played an important role in the process. Lake Qinghai contains relatively high plankton densities, which may have promoted the evolution of the densely rakered *G. p. przewalskii* instead of the sparsely rakered Yellow River *G. e. eckloni*. In contrast, the Ganzi River and Lake Keluke both provide abundant zoobenthos and algae. These trophic specializations resulted in morphological innovation through their effects on trophically-related traits. The trophic niches of the Ganzi River and Lake Keluke were comparable to that of the Yellow River *G. e. eckloni*, which may have encouraged the rapid adaptation of gill raker traits toward the ancestral state. Interestingly, the highest genetic differentiation was observed between the two youngest lineages, the Lake Keluke and Ganzi River lineages (*F*st = 0.6754; Table 4), indicating an earlier divergence from different ancestral lineages. However, the similarities between their phenotypes might also suggest analogous evolutionary responses for local adaptation associated with ecological specialization.

Unexpectedly, average gill raker numbers among the Lake Keluke *Gymnocypris* fish were substantially lower (17.91 for the outer rakers and 30.71 for the inner rakers of the first gill) than those previously described (31.29 and 48.71) by Wu and Wu [32]. This earlier study, which was based on seven individuals, did not demonstrate clear differences in external characters and gill rakers between the Lake Keluke population and that of Lake Qinghai; therefore, the Lake Keluke fish were included in the *G. p. przewalskii* subspecies [32]. In the present study, we observed gill raker counts completely different from those of *G. p. przewalskii*, and the molecular data supported the separation of the Lake Keluke *Gymnocypris* fish into a monophyletic sister group to the Lake Qinghai *G. p. przewalskii*. A comprehensive morphological and ecological analysis based on a more extensive sample of Lake Keluke *Gymnocypris* fish will be necessary to determine the appropriate taxonomic status of this population.

Lineage C contained all four morphs, suggesting that the greatest morphological diversity was associated with recent evolutionary events. Indeed, the rate of divergence was rapid among the morphs as early as 0.017 Ma. This observation implies that these species have a recent origin of divergence and an extreme sensitivity to environmental change. They also exhibit a great ability to adapt to different habitats.

The high genetic divergence of the three Yellow River *G. e. eckloni* lineages reflects their ancient history, but no consistent morphological differentiation within this population has been recorded thus far [32], [33]. This result suggests that the similarity of traits among the *G. e. eckloni* lineages is not due to insufficient time for divergence. We hypothesize that stable selection pressures and homogeneous conditions within the water system explain this observed pattern; further research is necessary to confirm this hypothesis.

The results of this study suggested a rapid evolutionary radiation with a tendency toward the ancestral phenotype in these *Gymnocypris* fish. Our study provides the first genetic evidence for the occurrence of convergence or reversal in the schizothoracine fish of the Tibetan Plateau at small temporal scales and highlights the need for a detailed examination of additional species’ phenotypic trajectories along a phylogeny to test the generality of the observed patterns.

## Supporting Information

Appendix S1 **Species and population distribution of haplotypes (ha).**
(DOC)
