# Supplementary material for: Ongoing Speciation in the Tibetan Plateau Gymnocypris Species Complex
Source: PLoS One. 2013 Aug 15;8(8):e71331. doi: 10.1371/journal.pone.0071331 (PMC3744573; doi:10.1371/journal.pone.0071331)
Supplement: Appendix S1 — Species and population distribution of haplotypes (ha). (DOC) [file pone.0071331.s001.doc]

Appendix S1. Species and population distribution of haplotypes (ha).

| ha | N | Species | Clade | Population (N) | | | GenBank Accession No. | | |
| --- | --- | --- | --- | --- | --- | --- | --- | --- | --- |
|  |  |  |  |  |  |  | Cyt *b* | 16S rRNA | RAG-2 |
| 1 | 2 | *G*. *p. przewalakii* | C | PQ (2) |  |  | KC733957 | KC733894 | KC733919 |
| 2 | 1 | *G*. *p. przewalakii* | C | PQ (1) |  |  | KC733957 | KC733894 | KC733920 |
| 3 | 1 | *G*. *p. przewalakii* | C | PQ (1) |  |  | KC733957 | KC733895 | KC733920 |
| 4 | 1 | *G*. *p. przewalakii* | C | PQ (1) |  |  | KC733957 | KC733894 | KC733921 |
| 5 | 1 | *G*. *p. przewalakii* | C | PQ (1) |  |  | KC733958 | KC733894 | KC733919 |
| 6 | 1 | *G*. *p. przewalakii* | C | PQ (1) |  |  | KC733957 | KC733894 | KC733922 |
| 7 | 1 | *G*. *p. przewalakii* | C | PQ (1) |  |  | KC733959 | KC733894 | KC733922 |
| 8 | 1 | *G*. *p. przewalakii* | C | PQ (1) |  |  | KC733959 | KC733894 | KC733923 |
| 9 | 1 | *G*. *p. przewalakii* | C | PQ (1) |  |  | KC733957 | KC733894 | KC733924 |
| 10 | 1 | *G*. *p. przewalakii* | C | PQ (1) |  |  | KC733957 | KC733894 | KC733925 |
| 11 | 1 | *G*. *p. przewalakii* | C | PQ (1) |  |  | KC733957 | KC733894 | KC733926 |
| 12 | 1 | *G*. *p. przewalakii* | C | PQ (1) |  |  | KC733957 | KC733896 | KC733927 |
| 13 | 1 | *G*. *p. przewalakii* | C | PQ (1) |  |  | KC733960 | KC733896 | KC733927 |
| 14 | 1 | *G*. *p. przewalakii* | C | PQ (1) |  |  | KC733961 | KC733896 | KC733927 |
| 15 | 1 | *G*. *p. przewalakii* | C | PQ (1) |  |  | KC733957 | KC733896 | KC733928 |
| 16 | 1 | *G*. *p. przewalakii* | C | PQ (1) |  |  | KC733962 | KC733894 | KC733929 |
| 17 | 1 | *G*. *p. przewalakii* | C | PQ (1) |  |  | KC733963 | KC733894 | KC733930 |
| 18 | 5 | *G*. *p. przewalakii* | C | PQ (5) |  |  | KC733959 | KC733894 | KC733927 |
| 19 | 1 | *G*. *p. przewalakii* | C | PQ (1) |  |  | KC733959 | KC733894 | KC733931 |
| 20 | 1 | *G*. *p. przewalakii* | C | PQ (1) |  |  | KC733964 | KC733894 | KC733927 |
| 21 | 1 | *G*. *p. przewalakii* | C | PQ (1) |  |  | KC733959 | KC733894 | KC733929 |
| 22 | 1 | *G*. *p. przewalakii* | C | PQ (1) |  |  | KC733957 | KC733894 | KC733932 |
| 23 | 1 | *G*. *p. przewalakii* | C | PQ (1) |  |  | KC733957 | KC733894 | KC733933 |
| 24 | 1 | *G*. *p. przewalakii* | C | PQ (1) |  |  | KC733957 | KC733894 | KC733934 |
| 25 | 1 | *G*. *p. przewalakii* | C | PQ (1) |  |  | KC733957 | KC733894 | KC733935 |
| 26 | 1 | *G*. *p. przewalakii* | C | PQ (1) |  |  | KC733957 | KC733894 | KC733936 |
| 27 | 1 | *G*. *p. przewalakii* | C | PQ (1) |  |  | KC733965 | KC733894 | KC733927 |
| 28 | 13 | *G*. *p. przewalakii* | C | PQ (13) |  |  | KC733957 | KC733894 | KC733927 |
| 29 | 7 | *G*. *p. przewalakii* | C | PQ (7) |  |  | KC733966 | KC733897 | KC733927 |
| 30 | 1 | *G*. *p. przewalakii* | C | PQ (1) |  |  | KC733967 | KC733897 | KC733927 |
| 31 | 1 | *G*. *p. przewalakii* | C | PQ (1) |  |  | KC733966 | KC733897 | KC733937 |
| 32 | 1 | *G*. *p. przewalakii* | C | PQ (1) |  |  | KC733966 | KC733898 | KC733927 |
| 33 | 1 | *G*. *p. przewalakii* | C | PQ (1) |  |  | KC733966 | KC733897 | KC733936 |
| 34 | 1 | *G*. *p. przewalakii* | C | PQ (1) |  |  | KC733966 | KC733897 | KC733938 |
| 35 | 1 | *G*. *p. przewalakii* | C | PQ (1) |  |  | KC733968 | KC733897 | KC733927 |
| 36 | 1 | *G*. *p. przewalakii* | C | PQ (1) |  |  | KC733969 | KC733897 | KC733927 |
| 37 | 1 | *G*. *p. przewalakii* | C | PQ (1) |  |  | KC733970 | KC733897 | KC733927 |
| 38 | 1 | *G*. *p. przewalakii* | C | PQ (1) |  |  | KC733970 | KC733897 | KC733939 |
| 39 | 3 | *G*. *p. przewalakii* | C | PQ (3) |  |  | KC733971 | KC733899 | KC733927 |
| 40 | 1 | *G*. *p. przewalakii* | C | PQ (1) |  |  | KC733971 | KC733899 | KC733935 |
| 41 | 1 | *G*. *p. przewalakii* | C | PQ (1) |  |  | KC733972 | KC733899 | KC733927 |
| 42 | 1 | *G*. *p. przewalakii* | C | PQ (1) |  |  | KC733972 | KC733899 | KC733928 |
| 43 | 1 | *G*. *p. przewalakii* | C | PQ (1) |  |  | KC733973 | KC733899 | KC733939 |

Appendix S1 (continued)

| ha | N | Species | Clade | Population (N) | | | GenBank Accession No. | | |
| --- | --- | --- | --- | --- | --- | --- | --- | --- | --- |
|  |  |  |  |  |  |  | Cyt *b* | 16S rRNA | RAG-2 |
| 44 | 1 | *G*. *p. przewalakii* | C | PQ (1) |  |  | KC733971 | KC733899 | KC733923 |
| 45 | 1 | *G*. *p. przewalakii* | C | PQ (1) |  |  | KC733971 | KC733899 | KC733940 |
| 46 | 1 | *G*. *p. przewalakii* | C | PQ (1) |  |  | KC733971 | KC733900 | KC733927 |
| 47 | 5 | *G*. *p. przewalakii* | C | PQ (5) |  |  | KC733974 | KC733897 | KC733927 |
| 48 | 1 | *G*. *p. przewalakii* | C | PQ (1) |  |  | KC733975 | KC733901 | KC733927 |
| 49 | 1 | *G*. *p. przewalakii* | C | PQ (1) |  |  | KC733976 | KC733897 | KC733927 |
| 50 | 1 | *G*. *p. przewalakii* | C | PQ (1) |  |  | KC733976 | KC733897 | KC767659 |
| 51 | 1 | *G*. *p. przewalakii* | C | PQ (1) |  |  | KC733977 | KC733897 | KC733927 |
| 52 | 1 | *G*. *p. przewalakii* | C | PQ (1) |  |  | KC733977 | KC733897 | KC733940 |
| 53 | 1 | *G*. *p. przewalakii* | C | PQ (1) |  |  | KC733978 | KC733897 | KC733927 |
| 54 | 1 | *G*. *p. przewalakii* | C | PQ (1) |  |  | KC733978 | KC733902 | KC733927 |
| 55 | 1 | *G*. *p. przewalakii* | C | PQ (1) |  |  | KC733974 | KC733897 | KC733940 |
| 56 | 1 | *G*. *p. przewalakii* | C | PQ (1) |  |  | KC733974 | KC733897 | KC733934 |
| 57 | 1 | *G*. *p. przewalakii* | C | PQ (1) |  |  | KC733979 | KC733897 | KC733942 |
| 58 | 39 | *G. p. ganzihonensis* | C | PG (39) |  |  | KC733980 | KC733903 | KC767651 |
| 59 | 2 | *G. p. ganzihonensis* | C | PG (2) |  |  | KC733980 | KC733903 | KC733943 |
| 60 | 2 | *G. p. ganzihonensis* | C | PG (2) |  |  | KC733980 | KC733903 | KC733944 |
| 61 | 1 | *G. p. ganzihonensis* | C | PG (1) |  |  | KC733981 | KC733903 | KC767651 |
| 62 | 2 | *G. p. ganzihonensis* | C | PG (2) |  |  | KC733982 | KC733903 | KC767651 |
| 63 | 2 | *G. p. ganzihonensis* | C | PG (2) |  |  | KC733980 | KC733904 | KC767651 |
| 64 | 14 | *G. p. ganzihonensis* | C | PG (14) |  |  | KC733980 | KC733903 | KC767656 |
| 65 | 1 | *G. p. ganzihonensis* | C | PG (1) |  |  | KC733980 | KC733903 | KC767649 |
| 66 | 27 | *G*. *p. przewalakii* | C | PK (27) |  |  | KC733957 | KC733905 | KC733927 |
| 67 | 1 | *G*. *p. przewalakii* | C | PK (1) |  |  | KC733983 | KC733905 | KC733927 |
| 68 | 7 | *G*. *p. przewalakii* | C | PK (7) |  |  | KC733957 | KC733905 | KC733931 |
| 69 | 7 | *G*. *p. przewalakii* | C | PK (7) |  |  | KC733957 | KC733905 | KC733933 |
| 70 | 1 | *G*. *p. przewalakii* | C | PK (1) |  |  | KC733957 | KC733905 | KC733945 |
| 71 | 4 | *G*. *p. przewalakii* | C | PK (4) |  |  | KC733957 | KC733905 | KC733946 |
| 72 | 2 | *G*. *p. przewalakii* | C | PK (2) |  |  | KC733957 | KC733905 | KC733947 |
| 73 | 2 | *G*. *p. przewalakii* | C | PK (2) |  |  | KC733957 | KC733905 | KC733923 |
| 74 | 10 | *G*. *e*. *eckloni* | C | EY2 (5) | EY4 (5) |  | KC733984 | KC757127 | KC767652 |
| 75 | 3 | *G*. *e*. *eckloni* | C | EY3 (2) | EY4 (1) |  | KC733984 | KC733906 | KC767652 |
| 76 | 1 | *G*. *e*. *eckloni* | C | EY3 (1) |  |  | KC733984 | KC733906 | KC733948 |
| 77 | 1 | *G*. *e*. *eckloni* | C | EY4 (1) |  |  | KC733984 | KC733906 | KC767653 |
| 78 | 1 | *G*. *e*. *eckloni* | C | EY1 (1) |  |  | KC733984 | KC733907 | KC767652 |
| 79 | 1 | *G*. *e*. *eckloni* | C | EY3 (1) |  |  | KC733985 | KC733906 | KC767650 |
| 80 | 1 | *G*. *e*. *eckloni* | C | EY2 (1) |  |  | KC733986 | KC757127 | KC767652 |
| 81 | 1 | *G*. *e*. *eckloni* | C | EY4 (1) |  |  | KC733986 | KC757127 | KC767658 |
| 82 | 1 | *G*. *e*. *eckloni* | C | EY1 (1) |  |  | KC733984 | KC757127 | KC733941 |
| 83 | 1 | *G*. *e*. *eckloni* | C | EY4 (1) |  |  | KC733984 | KC757127 | KC767655 |
| 84 | 1 | *G*. *e*. *eckloni* | C | EY2 (1) |  |  | KC733984 | KC757127 | KC767653 |
| 85 | 9 | *G*. *e*. *eckloni* | B | EY1 (4) | EY2 (3) | EY3 (2) | KC733987 | KC733908 | KC733941 |
| 86 | 1 | *G*. *e*. *eckloni* | B | EY1 (1) |  |  | KC733987 | KC733908 | KC767653 |

Appendix S1 (continued)

| ha | N | Species | Clade | Population (N) | | | GenBank Accession No. | | |
| --- | --- | --- | --- | --- | --- | --- | --- | --- | --- |
|  |  |  |  |  |  |  | Cyt *b* | 16S rRNA | RAG-2 |
| 87 | 3 | *G*. *e*. *eckloni* | B | EY1 (3) |  |  | KC733988 | KC733909 | KC767652 |
| 88 | 1 | *G*. *e*. *eckloni* | B | EY1 (1) |  |  | KC733989 | KC733909 | KC767657 |
| 89 | 1 | *G*. *e*. *eckloni* | B | EY1 (1) |  |  | KC733990 | KC733908 | KC767652 |
| 90 | 1 | *G*. *e*. *eckloni* | B | EY2 (1) |  |  | KC733991 | KC733910 | KC767652 |
| 91 | 3 | *G*. *e*. *eckloni* | B | EY3 (2) | EY4 (1) |  | KC733988 | KC733908 | KC767652 |
| 92 | 1 | *G*. *e*. *eckloni* | B | EY4 (1) |  |  | KC733992 | KC733908 | KC767655 |
| 93 | 1 | *G*. *e*. *eckloni* | B | EY4 (1) |  |  | KC733991 | KC733910 | KC733949 |
| 94 | 1 | *G*. *e*. *eckloni* | B | EY4 (1) |  |  | KC733991 | KC733910 | KC767654 |
| 95 | 1 | *G*. *e*. *eckloni* | B | EY3 (1) |  |  | KC733988 | KC733908 | KC733950 |
| 96 | 1 | *G*. *e*. *eckloni* | B | EY3 (1) |  |  | KC733993 | KC733908 | KC767652 |
| 97 | 1 | *G*. *e*. *eckloni* | B | EY4 (1) |  |  | KC733994 | KC733908 | KC767655 |
| 98 | 1 | *G*. *e*. *eckloni* | B | EY1 (5) |  |  | KC733987 | KC733908 | KC767652 |
| 99 | 13 | *G*. *e*. *eckloni* | A | EY1 (5) | EY2 (6) | EY3 (2) | KC733995 | KC733911 | KC767652 |
| 100 | 1 | *G*. *e*. *eckloni* | A | EY3 (1) |  |  | KC733996 | KC733912 | KC767652 |
| 101 | 1 | *G*. *e*. *eckloni* | A | EY3 (1) |  |  | KC733997 | KC733913 | KC733941 |
| 102 | 1 | *G*. *e*. *eckloni* | A | EY4 (1) |  |  | KC733995 | KC733914 | KC733951 |
| 103 | 1 | *G*. *e*. *eckloni* | A | EY4 (1) |  |  | KC733998 | KC733911 | KC767652 |
| 104 | 1 | *G*. *e*. *eckloni* | A | EY4 (1) |  |  | KC733995 | KC733915 | KC767652 |
| 105 | 1 | *G*. *e*. *eckloni* | A | EY3 (1) |  |  | KC733999 | KC733911 | KC767652 |
| 106 | 2 | *G*. *e*. *eckloni* | A | EY3 (1) | EY4 (1) |  | KC733995 | KC733914 | KC767652 |
| 107 | 1 | *G*. *e*. *eckloni* | A | EY4 (1) |  |  | KC733997 | KC733911 | KC733952 |
| 108 | 1 | *G*. *e*. *eckloni* | A | EY4 (1) |  |  | KC733997 | KC733911 | KC767655 |
| 109 | 1 | *G*. *e*. *eckloni* | A | EY4 (1) |  |  | KC733997 | KC733911 | KC733953 |
| 110 | 1 | *G*. *e*. *eckloni* | A | EY3 (1) |  |  | KC733997 | KC733911 | KC767652 |
| 111 | 1 | *G*. *e*. *chilianensis* |  |  |  |  | KC734000 | KC733916 | KC733954 |
| 112 | 1 | *G. waddelli* |  |  |  |  | KC734001 | KC733917 | KC733955 |
| 113 | 1 | *G. potanini* |  |  |  |  | KC734002 | KC733918 | KC733956 |
